# Pillar-Layered Metal-Organic Frameworks for Sensing Specific Amino Acid and Photocatalyzing Rhodamine B Degradation

**DOI:** 10.3390/molecules27217551

**Published:** 2022-11-03

**Authors:** Zi-Qing Huang, Shu-Man Zhao, Jia-Qi Chen, Yue Zhao, Wei-Yin Sun

**Affiliations:** Coordination Chemistry Institute, State Key Laboratory of Coordination Chemistry, School of Chemistry and Chemical Engineering, Nanjing National Laboratory of Microstructures, Collaborative Innovation Center of Advanced Microstructures, Nanjing University, Nanjing 210023, China

**Keywords:** metal-organic frameworks, amino acid, Rhodamine B, sensing, photodegradation

## Abstract

Metal-organic frameworks (MOFs) have presented potential for detection of specific species and catalytic application due to their diverse framework structures and functionalities. In this work, two novel pillar-layered MOFs [Cd_6_(DPA)_2_(NTB)_4_(H_2_O)_4_]_n_·n(DPA·5DMA·H_2_O) (**1**) and [Cu_2_(DPA)(OBA)_2_]_n_·n(2.5DMF·H_2_O) (**2**) [DPA = 2,5-di(pyridin-4-yl)aniline, H_3_NTB = 4,4′,4′′-nitrilotribenzoic acid, H_2_OBA = 4,4′-oxydibenzoic acid, DMA = N,N-dimethylacetamide, DMF = N,N-dimethylformamide] were successfully synthesized and structurally characterized. Both **1** and **2** have three-dimensional framework structures. The fluorescent property of **1** makes it possible for sensing specific amino acid such as L-glutamic acid (Glu) and L-aspartic acid (Asp). While MOF **2** was found to be suitable for photocatalytic degradation of Rhodamine B (RhB) in the presence of H_2_O_2_. The results imply that MOFs are versatile and metal centers are important in determining their properties.

## 1. Introduction

Nowadays, it attracts great attention with relation to the healthy and environmental issue such as detecting specific species and degrading harmful pollutants [1,2]. The detection of definite harmful species such as nitroaromatic compounds (NACs), ketone molecules, halogen flame retardants and so on have been extensively explored, however, the study on sensing biomolecules like amino acids (AAs) is limited [3,4,5,6]. It is known that AAs play key role in varied physiological activities [7,8]. Among them, L-glutamic acid (Glu) and L-aspartic acid (Asp) are important biological neurotransmitters, but may arise undesired side effects when their content exceeds the standard. For example, excessive Glu may lead to mental diseases such as Parkinson’s syndrome and allergic reactions like headache and nausea [9]. Therefore, the accurate detection of the AA is meaningful for monitoring and diagnosing human health.

Among the reported studies, luminescent metal-organic frameworks (MOFs) have been recognized as efficient and versatile detector due to their variable responses to the analytes including the change of luminant color, enhancement or quenching the fluorescence [10,11]. Besides, the unique porous structure may be helpful for adsorbing target analytes which may be selectively interacting with the framework by the porous skeleton [12,13]. In our previous work, it was found that the amino-functionalized MOF, namely NH_2_-MIL-101, can be utilized for sensing specific AA in aqueous media via turn-on fluorescence [14]. In addition to the detection, MOFs have also been widely employed in photocatalysis, like water splitting, CO_2_ reduction, organic reactions and so on [15,16,17,18,19,20]. The removal of organic pollutants in the waste water is a significant project with the methods like adsorption and in situ degradation [21,22,23,24,25,26,27]. Among the common pollutants, organic dyes are widely utilized in industrial production, which is in large dosage and strong persistence in the environment resulting in harm to human kidneys and organs [28]. Rhodamine B (RhB) is well used and has been warned in the food industry due to its carcinogenicity and neurotoxicity [29,30]. Therefore, efficient removal of RhB is essential and photocatalytic degradation is an efficient and environment-friendly approach. It has been reported that the assistance of H_2_O_2_ is critical to the degradation of organic dyes by using Fenton-like catalysts such as iron-based oxides or complex [31]. In this work, a Cu(II) pillar-layered MOF was applied for photocatalizing degradation of RhB.

## 2. Results and Discussion

### 2.1. Crystal Structure Description of MOF ***1***

**Figure 1:**
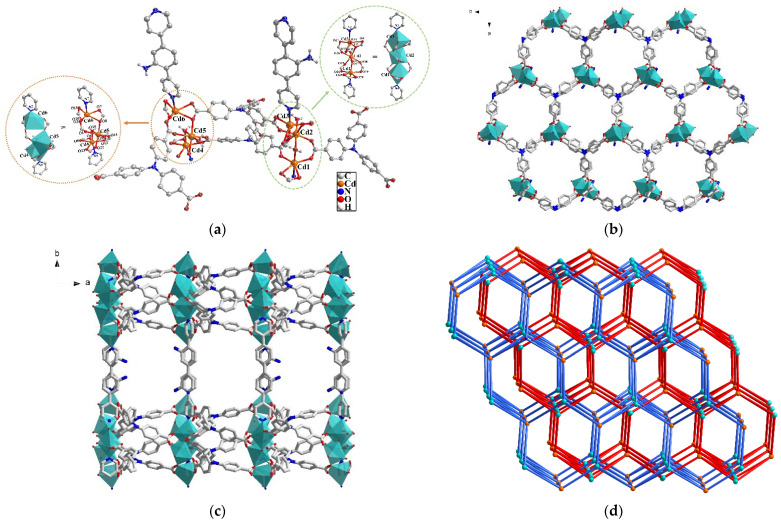
(**a**) Coordination environment of Cd(II) in **1** with 50% thermal ellipsoidal ratio, and the hydrogen atoms and lattice molecules are omitted. (**b**) 2D network in **1**. (**c**) 3D pillar-layered framework viewed along c-axis. (**d**) The two-fold interpenetration and topology of **1**.

Single crystal X-ray diffraction (SC-XRD) data present that **1** crystallizes in monoclinic space group of *P*2_1_/c (Table 1). As illustrated in Figure 1a, the repeating unit is constructed by six Cd(II) cations, four deprotonated anions of NTB^3−^, two neutral ligands DPA and four coordinated H_2_O molecules. In addition, there are one DPA, five DMA and one water as lattice molecules in the voids of **1**. There are two different trinuclear secondary building units (SBUs) containing Cd1, Cd2, Cd3 and Cd4, Cd5, Cd6, respectively (Figure 1a,b). Each Cd(II) is six-coordinated with distorted octahedral coordination geometry. For example, Cd1 is surrounded by four oxygen atoms (O16, O19, O28, O29) belonging to three carboxylate of NTB^3−^, one water molecule (O17) and one nitrogen atom (N4) from DPA. Cd2 is coordinated by six oxygen atoms from two water molecules (O17 and O24) and four carboxylate ones (O3, O15, O18, O26) of four different NTB^3−^. While Cd3 is bound by five oxygen atoms (O3, O4, O13, O14 and O27) from three carboxylate NTB^3−^ and one nitrogen one (N2) from DPA. Cd4, Cd5 and Cd6 atoms have similar coordination environment with the Cd1, Cd2 and Cd3, respectively. It is noteworthy that Cd1 and Cd2 as well as Cd4 and Cd5 are linked together by a water molecule as bridging ligand and two carboxylate groups, while the Cd2 and Cd3 as well as Cd5 and Cd6 are joined together by one oxygen atom from a carboxylate and another carboxylate group to form the Cd_3_ trinuclear SBU, respectively. There are two coordination modes of (μ_1_-η^1^:η^1^)-(μ_2_-η^2^:η^1^)-(μ_2_-η^1^:η^1^) and (μ_1_-η^1^:η^1^)-(μ_2_-η^1^: η^1^)-(μ_2_-η^1^:η^1^) for NTB^3−^ in **1** (Figure S2). The SBUs are connected by NTB^3−^ to extend into a two-dimensional (2D) network (Figure 1b), which is further linked by DPA as two-connected pillar to generate a three-dimensional (3D) framework of **1** with pillar-layered structure (Figure 1c) [32,33]. Furthermore, the final structure of **1** has two-fold interpenetration and the topological calculation (Figure 1d) was conducted to simplify the structure of **1** by regarding the Cd_3_ trinuclear SBU as eight- and NTB^3−^ as three-connected nodes. As a result, **1** is a (3,8)-connected two-node net with topological notation as {4^3^·6^24^·8}{4^3^}_2_.

### 2.2. Crystal Structure Description of MOF ***2***

**Figure 2:**
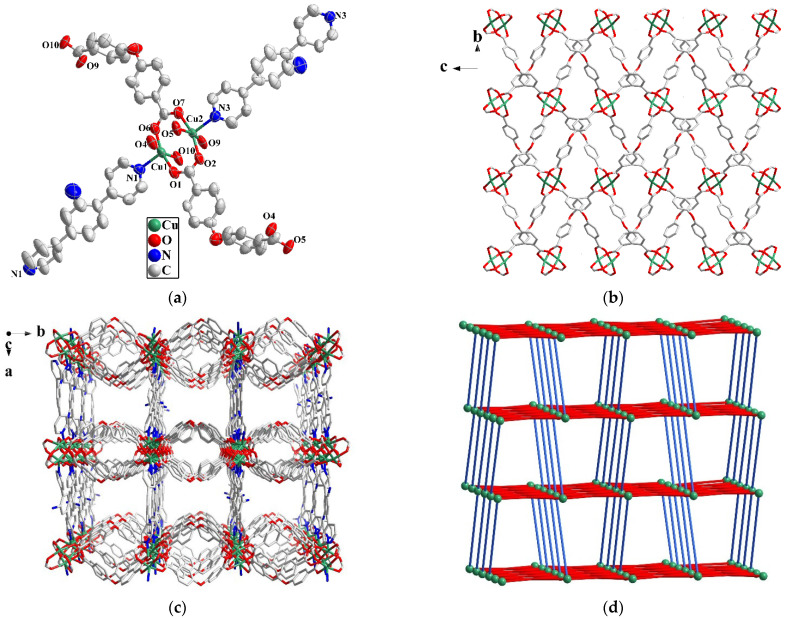
(**a**) The coordination environment of Cu(II) in **2** with 50% thermal ellipsoidal ratio, and the hydrogen atoms are omitted. (**b**) Cu-OBA^2−^ 2D network in **2**. (**c**) 3D pillar-layered structure of **2** viewed along c-axis. (**d**) Topology of **2**.

When H_2_OBA was used instead of H_3_NTB, and CuI and KI were added in the reaction, Cu-MOF **2**, rather than a Cu-Cd bimetallic MOF, was achieved. **2** crystallizes in orthorhombic space group *P*bcn (Table 1). The repeat unit has two Cu(II), one DPA and two OBA^2−^ (Figure 2a). Each Cu(II) is five-coordinated with four oxygen atoms from four different carboxylate groups of OBA^2−^ and a nitrogen from DPA. Two Cu(II) and four carboxylate groups of OBA^2−^ form a [Cu_2_(COO)_4_] paddle wheel-like SBU, which is extended into a 2D network by the connection of OBA^2−^ (Figure 2b). The 2D layers are further connected by DPA to form a 3D framework with the pillar-layered structure (Figure 2c). The pore volume in **2** is calculated to be 1259.9 Å^3^ (34.7%) by PLATON after removing the solvent molecules. The Brunauer Emmett Teller (BET) surface area of MOF **2** is 136.31 m^2^/g determined by N_2_ adsorption data at 77 K (Figure S3). Considering the SBU as a six-connected node and the ligand as a linear linker, the topology of **2** can be simplified to be {4^4^·6^10^·8} with a 1D channel (Figure 2d).

### 2.3. Powder X-ray Diffraction (PXRD) and Thermogravimetric Analyses (TGA)

PXRD data were utilized to ensure the phase purity of the as-synthesized samples **1** and **2**. As shown in Figure S4, the characteristic diffraction peaks of the as-synthesized samples are consistent with the simulated ones, which imply that the synthesized samples are in pure phase. The thermal stability of the MOFs was estimated by TG measurements under N_2_ atmosphere. As shown in Figure S5, gradual weight loss of ca. 19% in **1** was observed before 350 °C, which is caused by release of terminal water and lattice molecules (Calcd. 21.4%). The collapse of the framework of **1** starts from 360 °C. As for **2**, weight loss of 18.2% was detected in the range of 25–195 °C, which is corresponding to the loss of DMF and water molecules (Calcd. 18.5%). The framework was maintained until 325 °C.

### 2.4. Stability of MOF ***1*** in Different Solvent

It is known that the practical application of MOFs is dependent on their stability [34]. Accordingly, the structural stability of **1** in different solvent was tested by PXRD. As exhibited in Figure S6, the as-synthesized **1** was respectively immerged in varied solvent including water, methanol (MeOH), ethanol (EtOH), acetonitrile, DMF, DMA, dichloromethane (DCM) and isopropanol (IPA). The PXRD patterns were almost maintained in these medium, except for the one in MeOH with slight disturbance. The high stability of **1** may be ascribed to the two-fold interpenetration [35,36].

### 2.5. Photoluminescence of MOF ***1***

It has been recognized that MOFs with d^10^ metal centers and π-conjugated organic ligands may possess photoluminescence (PL) [37]. Thus, MOF **1** with 3d^10^ metal nodes of Zn(II) may show PL. However, no PL can be expected for MOF **2** due to the 3d^9^ Cu(II) centers, instead photocatalytic property of MOF **2** was tested (vide post). The PL of MOF **1** as well as H_3_NTB and DPA ligands was examined in the solid state at room temperature. As illustrated in Figure S7, the emission of **1** at 487 nm (λ_ex_ = 383 nm) may mainly arise from the ligand H_3_NTB since H_3_NTB gives an emission at 460 nm (λ_ex_ = 397 nm). The red-shift and enhancement of the emission in **1** is probably caused by the coordination between the Cd(II) and NTB^3−^ to increase the rigidity of the framework [38]. In addition, DPA shows negligible luminescence, which may be caused by the intramolecular resonance energy transfer (RET) and inner filter effect (IFE) due to presence of amino group [39,40]. To explore the influence of solvent on emission of **1** [41], PL spectra of **1** after immerging in varied solvent of DMF, EtOH, IPA, DMA, CH_3_CN, MeOH, toluene, DCM and H_2_O were recorded. As shown in Figure S8, **1** exhibits solvent dependent emission with different intensity and wavelength.

### 2.6. Fluorescence Sensing Specific AA by ***1***

**Figure 3:**
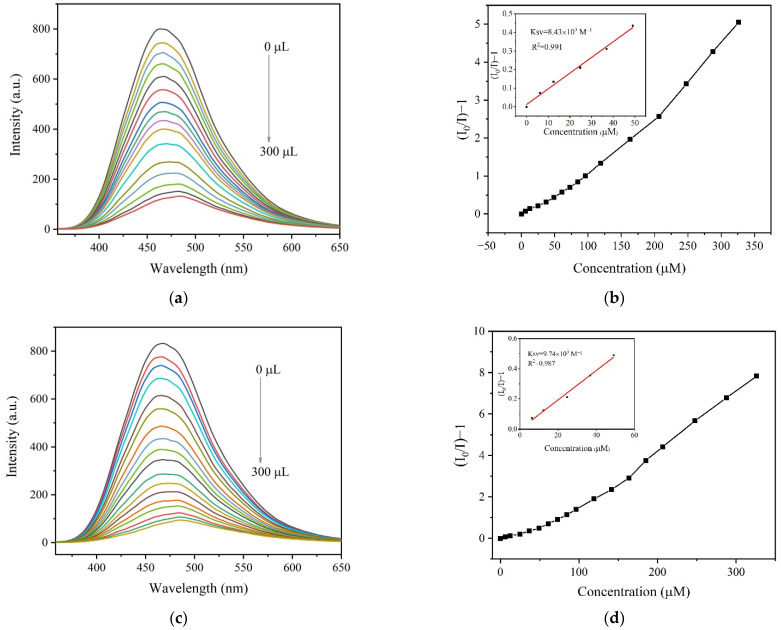
Fluorescence titration of **1** with gradual addition of Glu (**a**) and Asp (**c**). The S-V plot of I_0_/I − 1 vs. the concentration of Glu (**b**) and Asp (**d**). Inset: enlarged view of S-V plot in the low concentration range and the calculated quenching constant K*_sv_*.

The detection of specific AA is of great significance in nutritional conditioning and disease diagnosis [42]. In addition, water was employed as the detection medium since AAs generally exist in normal saline. The fluorescence sensing performance of **1** for specific AA was investigated in aqueous solution of L-tryptophan (Trp), L-tyrosine (Tyr), L-threonine (Thr), L-isoleucine (Ile), L-phenylalanine (Phe), L-alanine (Ala), L-serine (Ser), L-leucine (Leu), L-proline (Pro), L-histidine (His), glycine (Gly), L-valine (Val), L-methionine (Met), L-lysine (Lys), L-arginine (Arg), L-asparagine (Asn), L-glutamine (Gln), L-cysteine (Cys), Glu and Asp. As shown in Figure S9, the obvious quenching was detected in the aqueous solution of Glu as well as Asp, implying the sensing capacity of **1** for specific AA of Glu and Asp. Furthermore, the titration experiment was performed for reflecting the relationship between the fluorescence intensity and the concentration of the analyte (Figure 3). The linear Stern-Volmer (S-V) equation of I_0_/I = K*_sv_* [Q] + 1 was utilized, where I_0_ and I are the luminescence intensities before and after adding the analyte, Q is the molar concentration of the analyte, and K*_sv_* is quenching constant. As a result, the calculated K*_sv_* are 8.43 × 10^3^ M^−1^ for Glu and 9.74 × 10^3^ M^−1^ for Asp. The detection limits (DL) were determined according to the formula DL = 3 σ/K*_sv_* (σ is standard deviation) and the results are 4.44 × 10^−5^ M for Glu and 1.05 × 10^−4^ M for Asp (Table S2).

In order to clarify the mechanism of quenching process for **1** by Asp and Glu, PXRD and IR spectral measurements were conducted (Figure S10) and comparison for the samples before and after the detection was carried out. It can be seen that the PXRD patterns and IR spectra are almost the same with the original one, excluding the quenching caused by collapse of the framework structures. Furthermore, the RET and IFE effect were excluded by the non-overlapping between the PL spectra of **1** (360–650 nm) and the UV absorption of Asp and Glu (205 nm) (Figure S11). Therefore, the fluorescence quenching was considered to be in a static mode supported by the obvious increase of the fluorescence lifetime after detection (Figure S12 and Table S3) [43].

### 2.7. Photocatalytic Degradation of RhB by MOF ***2***

**Figure 4:**
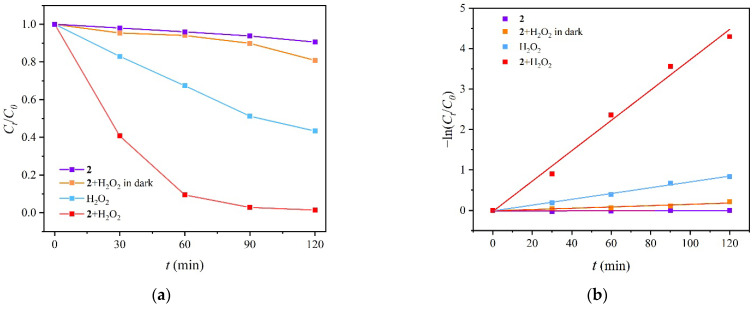
The concentration of RhB (**a**) and pseudo-first order kinetic curves (**b**) with varied reaction time.

RhB is commonly used in industry but toxic, thus it is necessary to completely remove RhB from the wastewater. In this study, MOF **2** was attempted to degrade RhB by photocatalysis with the assistance of H_2_O_2_. Firstly, the experimental standard curve (Figure S13) was fitted by the Lambert Beer law: Abs = KBC, where Abs is the absorbance of the tested mixture, K is the molar absorbance coefficient, B is the thickness of the total solution volume and C is the concentration of RhB in aqueous solution. Then, based on the external standard method, a series of C_t_/C_0_ photodegradation plots, where C_0_ is the initial concentration of RhB and C_t_ is the concentration at time t, were obtained with the varied reaction time (Figure 4) and the degradation efficiency was calculated by the formula (C_0_ − C_t_)/C_0_ [44]. It was found that high efficiency of 99% was achieved by combination of **2** and H_2_O_2_, which is satisfactory by comparing with the reported results (Figure S14 and Table S4) [45,46,47,48]. Besides, the contrast experiments were conducted to find out the influential factors. The efficiency was declined to 25% in the dark condition and the degradation was 13% and 56% when separately catalyzed by sole MOF **2** and H_2_O_2_. In addition, the concentration and degradation efficiency were examined at varied pH and it was found that the acidic environment is more suitable for the degradation (Figures S15 and S16), while in the basic environment the less degradation efficiency may be caused by the decomposition of H_2_O_2_ to O_2_ and H_2_O [31].

Based on the above results, the remarkable synergistic effect was present and the synergistic index (SI) was calculated by the photocatalytic degradation kinetics. As shown in Figure 4, the obtained data were fitted well with pseudo-first order in the formula ln (C_t_/C_0_) = kt, where k is the kinetic rate constant for quantitatively analyzing the photocatalytic performance. The constant k is 0.0375 min^−1^ in the synergistic system of **2** and H_2_O_2_, which is much larger than the one catalyzed by sole catalyst of **2** with negligible result and H_2_O_2_ with 0.007 min^−1^. Therefore, the SI was calculated to be ca. 5, according to the formula SI = k_(1+2)_/(k_1_ + k_2_).

**Figure 5:**
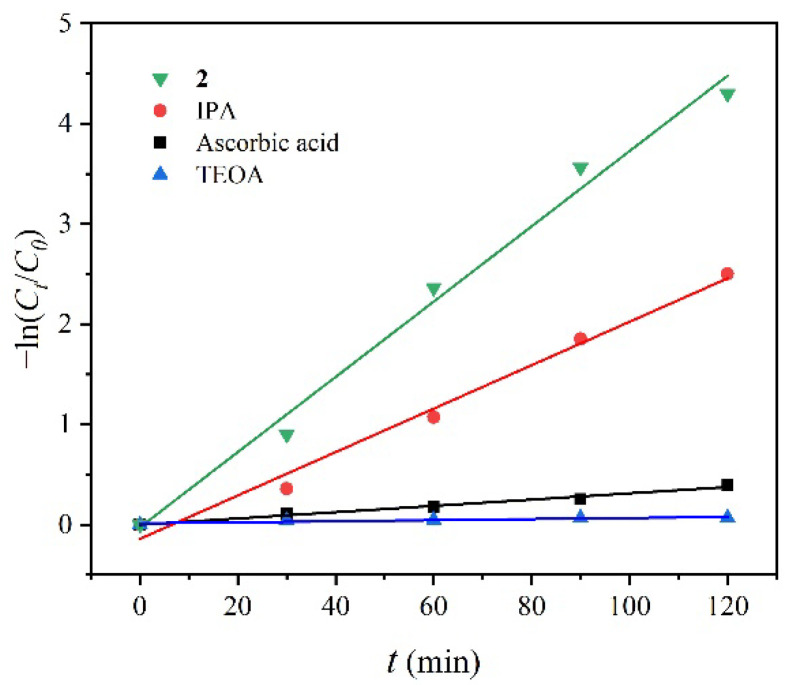
The pseudo-first order kinetic curves at varied photodegradation time with different radical trapping agents.

In order to analyse the photocatalytic degradation mechanism of RhB by **2**, the free radical capture experiment was carried out on the photocatalytic degradation process (Figure 5 and Figure S17). IPA, triethanolamine (TEOA) and ascorbic acid were respectively utilized as free radical trapping agents of hydroxyl radical ·OH, hole H^+^ and superoxide radical ·O^2−^ [49,50,51,52]. Among them, the capture of ·OH by IPA slightly reduced the degradation efficiency and the obvious inhibition was observed for hole-scavenger TEOA and superoxide scavenger ascorbic acid with k = 0.00317 and 0.0005 min^−1^, respectively, indicating that the H^+^ and ·O^2−^ play major role in the degradation of RhB. The reactive oxygen species (ROSs) produced in the degradation process at the first one hour were checked by EPR spectra with the assistance of 5,5-dimethyl-1-pyrroline N-oxide (DMPO) that acted as the spin trapping agent. It is obviously that the DMPO-·OH adduct was observed with the characteristic intensities of 1:2:2:1 (Figure S18), which supported the existence of hydroxyl radical at the initial stage of degradation [53].

**Figure 6:**
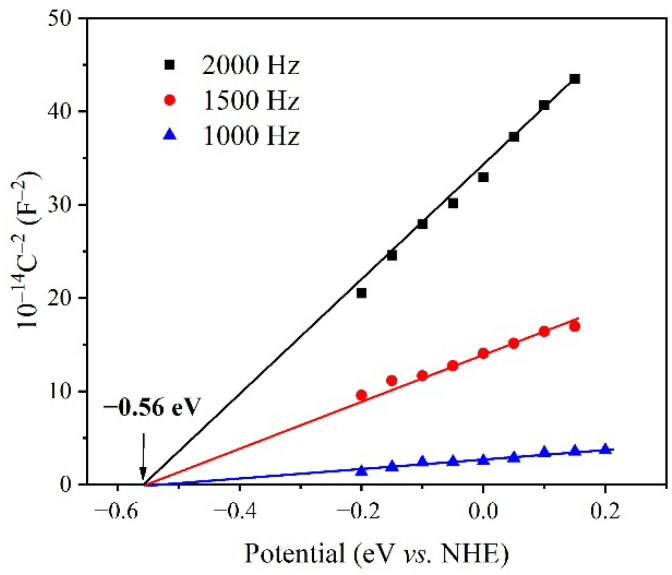
Mott Schottky plot of MOF **2** under varied frequencies.

The photocatalytic performance of MOFs is similar to that of semiconductor material, in which the electrons were transferred between the conduction band (CB) and valence band (VB). The solid UV-vis diffuse reflection spectrum of **2** shows two kinds of absorption bands in the range of 200–420 nm and 520–800 nm (Figure S19) [54]. Accordingly, the band gap of **2** was calculated to be 2.66 eV by the Tauc plot [15]. The conduction band potential, which is similar to the flat band position (VFB) of **2**, was measured by Mott Schottky experiments at varied frequencies of 1, 1.5 and 2 kHz. It can be seen from Figure 6 that the slope of the curve is positive, which shows that **2** has an n-type semiconductor character. Based on these results, it can be determined that the CB of **2** is −0.56 eV. Therefore, the valence band potential VB was calculated to be 2.10 eV by considering the band gap of 2.66 eV [15]. Furthermore, the EIS of MOF **2** was presented in Figure S20.

**Figure 7:**
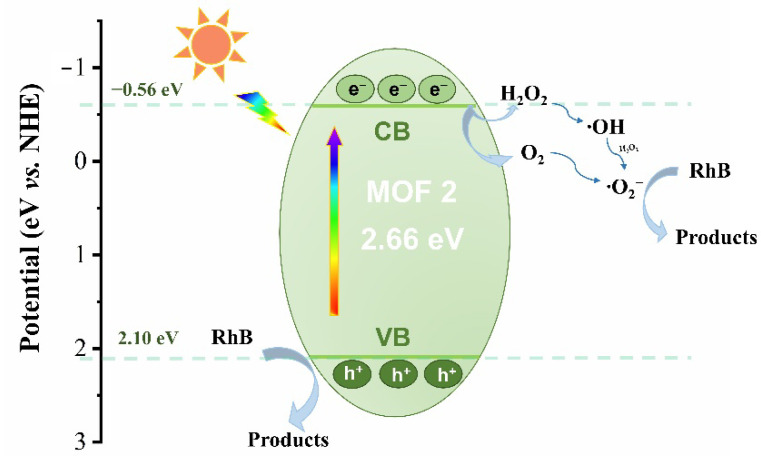
Possible mechanism of photodegradation of RhB by MOF **2** and H_2_O_2_.

Based on the above experimental results, the photocatalytic degradation mechanism is proposed (Figure 7). Since MOF **2** serves as an n-type semiconductor, electrons in the VB are excited to CB forming electron-hole pairs upon visible light irradiation. The photogenerated holes H^+^ in **2** were capable to directly react with RhB by the obvious attenuated efficiency after adding TEOA. As an electron acceptor, H_2_O_2_ was activated and produced ·OH (Figure S18), which meanwhile inhibits the recombination of electron-hole pairs for improving the photocatalytic performance of **2**. And the obtained hydroxyl radical ·OH was allowed to react with the excess H_2_O_2_ to generate ·O^2−^ [52]. In addition, the potential of CB is −0.56 eV in MOF **2**, which is more negative than the required potential −0.33 eV vs. NHE for reducing O_2_ to ·O^2−^. Thus, the oxygen existed in the solution or obtained by the decomposition of H_2_O_2_ was allowed to be reduced into ·O^2−^. Finally, the degradation of RhB occurred by the reaction with effective superoxide radical ·O^2−^.

## 3. Materials and Methods

### 3.1. Synthesis of [Cd_6_(DPA)_2_(NTB)_4_(H_2_O)_4_]_n_·n(DPA·5DMA·H_2_O) (***1***)

The reagents involved in the experiments were purchased commercially, DPA and H_3_NTB were prepared according to the previous literatures [32,55]. MOF **1** was prepared by mixing Cd(ClO_4_)_2_·6H_2_O (21.0 mg, 0.05 mmol), 4,4′,4′′-nitrilotribenzoic acid (H_3_NTB) (12.4 mg, 0.03 mmol), 2,5-di(pyridin-4-yl)aniline (DPA) (8.2 mg, 0.03 mmol) in a mixed solvent of N,N-dimethylacetamide (DMA) and H_2_O (3 mL, *v/v* = 2:1) in a 10 mL glass bottle, which was then heated at 100 °C for 3 days. Block crystals of **1** were obtained in about 88% yield based on H_3_NTB. Anal. Calcd. for C_152_H_142_N_18_O_34_Cd_6_: C, 53.08; H, 4.16; N, 7.33%. Found: C, 52.12; H, 4.21; N, 7.33%. IR (cm^−1^, ATR, Figure S1): 3431(w), 3358(w), 3235(w), 3057(w), 1589(s), 1538(m), 1503(m), 1382(s), 1313(m), 1272(m), 1172(m), 1101(w), 1073(w), 1013(m), 849(w), 811(w), 780(m).

### 3.2. Synthesis of [Cu_2_(DPA)(OBA)_2_]_n_·n(2.5DMF·H_2_O) (***2***)

For **2**, CuI (9.5 mg, 0.05 mmol), Cd(ClO_4_)_2_·6H_2_O (21.0 mg, 0.05 mmol), 4,4′-oxydibenzoic acid (H_2_OBA) (12.9 mg, 0.05 mmol), DPA (12.4 mg, 0.05 mmol), KI (10 mg, 0.06 mmol) and N,N-dimethylformamide (DMF) (3 mL) were mixed in a 10 mL glass bottle and then heated at 100 °C for 3 days. Block crystals of **2** were collected in 67% yield based on H_2_OBA. Anal. Calcd. for C_51.5_H_48.5_N_5.5_O_13.5_Cu_2_: C, 56.88; H, 4.50; N, 7.08%. Found: C, 55.90; H, 4.29; N, 7.21%. IR (cm^−1^, ATR, Figure S1): 3449(w), 3352(w), 3233(w), 3061(w), 1674(m), 1604(s), 1572(m), 1500(m), 1389(s), 1229(s), 1159(m), 1087 (m), 1011(w), 1073(w), 1013(m), 875(w), 798(w), 779(m).

### 3.3. Fluorescent Sensing AA by MOF ***1***

For sensing definite AA, the as-synthesized **1** was dispersed in H_2_O to produce a 0.5 mg mL^−1^ aqueous solution. All emission spectra were recorded in the range of 350–650 nm under excitation at 380 nm.

### 3.4. Photocatalyzing Degradation of RhB by MOF ***2***

MOF **2** (20 mg) and H_2_O_2_ solution (400 μL, 30%) were added into RhB aqueous solution (50 mL, 10 mg/L) and the mixture was pre-treated with stirring in the dark for 30 min. The degradation reaction was conducted under the visible light for **2** h by a 300 W xenon arc lamp with AM 1.5G filter serving as the light source. The reaction solution was taken 3 mL in every 30 min to centrifuge and measure its corresponding UV absorption spectrum.

### 3.5. X-ray Crystallography

Single-crystal X-ray diffraction data were collected on a Bruker D8 Venture diffractometer with graphite-monochromated Mo Kα radiation (λ = 0.71073 Å). The integration of diffraction data and intensity corrections for the Lorentz and polarization effects were performed by using SAINT program [56]. Semi-empirical absorption corrections were applied using SADABS program [57]. The structures were solved by direct methods with SHELXT-2014, expanded by subsequent Fourier-difference synthesis, and all the non-hydrogen atoms were refined anisotropically on *F*^2^ using the full-matrix least-squares technique with the SHELXL-2018 crystallographic software package [58,59]. Part of the free solvent molecules in **1** and the ones in **2** have been taken into account by SQUEEZE option of the PLATON program [60]. The details of crystal parameters, data collection and refinements for **1** and **2** are listed in Table 1, and the selected bond lengths and angles are given in Table S1. CCDC numbers 2,212,527 (for **1**) and 2,212,528 (for **2**) contain the supplementary crystallographic data for the reported compounds. These data can be obtained free of charge from The Cambridge Crystallographic Data Centre.

## 4. Conclusions

In this study, dipyridyl and multicarboxylate ligands were utilized to react with metal salts to generate 3D MOFs **1** and **2** with pillar-layered structure. The results show that **1** has high stability and presents distinct photoluminescence responses to the varied solvent. Furthermore, MOF **1** exhibits potential for sensing specific amino acid such as Glu and Asp through fluorescence quenching in the aqueous solution. In addition, MOF **2** has n-type semiconductor character and shows photocatalytic capacity for degradation of RhB in the presence of H_2_O_2_. The results of this study demonstrate the importance of the metal center in determining the property of the frameworks.

## Figures and Tables

**Table 1:** Crystal data and refinement results for **1** and **2**.

Compound	1	2
Formula	C_152_H_142_N_18_O_34_Cd_6_	C_51.5_H_48.5_N_5.5_O_13.5_Cu_2_
Formula weight	3439.23	1087.17
Crystal system	monoclinic	orthorhombic
Space group	*P*2_1_*/*c	*P*bcn
a (Å)	24.6399(11)	28.208(3)
b (Å)	22.0378(13)	23.453(2)
c (Å)	28.1864(13)	15.7977(16)
β (°)	90.107(2)	90
V (Å^3^)	15,305.4(13)	10,451.2(18)
Z	4	2
Dcalc (g·cm^−3^)	1.493	1.127
μ (mm^−1^)	0.898	0.863
F (000)	6960.0	3616.0
Reflections collected	120,401	78,324
Unique reflections	27,987	9561
Goodness-of-fit on *F*^2^	1.049	1.016
*R*_1_^a^, *wR*_2_^b^ [I > 2σ(*I*)]	0.0517/0.1351	0.1066/0.2760
*R*_1_, *wR*_2_ [all data]	0.0783/0.1613	0.1435/0.3023

^a^*R*_1_ = Σ||*F*_o_| − |*F*_c_||/Σ|*F*_o_|. ^b^ *wR*_2_ = |Σ*w*(|*F*_o_|^2^ − |*F*_c_|^2^)|/Σ|*w*(*F*_o_)^2^|^1/2^, where *w* = 1/[σ^2^(*F*_o_^2^) + (*aP*)^2^ + *bP*]. *P* = (*F*_o_^2^ + 2*F*_c_^2^)/3.

## Data Availability

Not applicable.

## Supplementary Materials

The following supporting information can be downloaded at: https://www.mdpi.com/article/10.3390/molecules27217551/s1, Table S1: Selected bond lengths (Å) and angles (°) for **1** and **2**; Table S2: Standard deviation and detection limit calculation of **1** for Glu and Asp in aqueous suspension. Table S3: Fluorescence lifetime of MOF **1** before and after addition of Glu and Asp. Table S4: H_2_O_2_-assisted photocatalytic degradation of aqueous RhB by MOF **2** and reported MOFs under visible light. Figure S1: FTIR-ATR spectra of **1** and **2**; Figure S2: Coordination modes of NTB^3−^ in **1**; Figure S3: The N2 adsorption isotherm of MOF **2** at 77 K; Figure S4: PXRD patterns of **1** and **2**; Figure S5: TG curves of **1** and **2**; Figure S6: PXRD of **1** after immerging in different solvent; Figure S7: Fluorescence spectra of MOF **1** and its ligands H_3_NTB and DPA in the solid state; Figure S8: PL spectra of **1** after immerging in varied solvent; Figure S9: Fluorescence quenching effect of **1** by adding amino acid aqueous solution (300 μL, 2.5 mM, λ_ex_ = 338 nm); Figure S10: PXRD and FTIR-ATR spectra of MOF **1** before and after detecting Glu and Asp; Figure S11: Fluorescence emission spectra of **1** and UV absorption spectra of Glu and Asp in water; Figure S12: Fluorescence lifetime of **1** before and after detecting Glu (a) and Asp (b) in water; Figure S13: The standard curve of MOF **2** for the UV absorption versus concentration of RhB; Figure S14: The degradation efficiency of RhB at varied reaction time; Figure S15: The concentration of RhB in the degradation process at acidic (pH = 2 and 5) and basic (pH = 8 and 10) conditions; Figure S16: The degradation efficiency of RhB by **2** at acidic (pH = 2 and 5) and basic (pH = 8 and 10) conditions. Figure S17: The concentration of RhB at varied photodegradation time with different radical trapping agents; Figure S18: The EPR spectra of photodegradation of RhB by MOF **2** at different reaction time using DMPO as spin-trapping agent; Figure S19: UV-vis diffuse reflectance spectrum (a) and Tauc plot (b) of MOF **2**; Figure S20: EIS Nyquist plot of MOF **2**.
