# Supplementary material for: Pillar-Layered Metal-Organic Frameworks for Sensing Specific Amino Acid and Photocatalyzing Rhodamine B Degradation
Source: Molecules. 2022 Nov 3;27(21):7551. doi: 10.3390/molecules27217551 (PMC9655496; doi:10.3390/molecules27217551)
Supplement: Supplementary file 1 [file molecules-27-07551-s001.zip › molecules-2006806-supplementary.pdf]

Supplementary Material

Pillar-Layered Metal-Organic Frameworks for Sensing Specific Amino Acid and Photocatalyzing Rhodamine B Degradation

Zi-Qing Huang†, Shu-Man Zhao†, Jia-Qi Chen, Yue Zhao and Wei-Yin Sun*

Coordination Chemistry Institute, State Key Laboratory of Coordination Chemistry, School of Chemistry and Chemical Engineering, Nanjing National Laboratory of Microstructures, Collaborative Innovation Center of Advanced Microstructures, Nanjing University, Nanjing 210023, China

* Correspondence: sunwy@nju.edu.cn

† These authors contributed equally to this work.

Materials and Methods

The reagents involved in the experiments were purchased commercially, DPA and H_3NTB were prepared according to the previous literatures [1,2]. Powder X-ray diffraction (PXRD) data were obtained on a Bruker D8 X-ray diffractometer with Cu Kα radiation (λ = 1.5418 Å). Element analysis (EA) data were acquired on the Elementar Vario MICRO cube elemental analyzer. FT-IR-ATR spectra were recorded on a Bruker Vector 22 FT-IR spectrometer in 4000-400 cm^{-1}. Thermogravimetric analyses (TGA) were performed on a Mettler-Toledo (TGA/DSC1) thermal analyzer under a nitrogen atmosphere with a heating rate of 10 °C/min. Fluorescence spectra (PL) were measured on a PerkinElmer LS-55 fluorescence spectrophotometer. Time-resolved fluorescence decay spectra were performed on a HORIBA Jobin Yvon FL-3 spectrometer. UV-vis spectra were captured on a Shimadzu UV-3600 spectrophotometer. The Mott-Schottky experiment was carried out on a Zahner electrochemical workstation in a standard three-electrode system, containing the sample coated glassy carbon as working electrode, a Pt wire as the counter electrode and a saturated calomel electrode as the reference electrode.

Table S1. Selected bond lengths (Å) and angles (°) for **1** and **2**.

	**1**		
Cd3-O3#1	2.289(4)	O24-Cd2-O26	94.99(16)
Cd3-O4#1	2.457(4)	O24-Cd2-O15	86.49(19)
Cd3-O27	2.248(4)	O15-Cd2-O3#1	166.99(16)
Cd3-O13#2	2.340(4)	O15-Cd2-O17	98.85(16)
Cd3-O14#2	2.331(4)	O15-Cd2-O26	88.90(16)
Cd3-N2	2.276(5)	O20#3-Cd6-N7	140.45(16)
Cd2-O3#1	2.337(4)	O20#3-Cd6-O8	103.24(15)
Cd2-O17	2.367(4)	O20#3-Cd6-O7	103.86(17)
Cd2-O18	2.204(4)	O20#3-Cd6-O21#3	54.80(15)
Cd2-O26	2.262(4)	N7-Cd6-O8	113.58(16)
Cd2-O24	2.229(4)	N7-Cd6-O7	87.15(18)
Cd2-O15	2.260(4)	N7-Cd6-O21#3	86.94(16)
Cd6-O20#3	2.268(4)	O8-Cd6-O21#3	139.05(16)
Cd6-N7	2.289(5)	O7-Cd6-O8	54.20(15)
Cd6-O8	2.420(4)	O7-Cd6-O21#3	94.51(17)
Cd6-O7	2.359(4)	O2-Cd6-O20#3	101.30(16)
Cd6-O2	2.242(4)	O2-Cd6-N7	88.80(17)
Cd6-O21#3	2.467(4)	O2-Cd6-O8	96.45(16)
Cd5-O10	2.395(4)	O2-Cd6-O7	144.93(17)
Cd5-O1	2.254(4)	O2-Cd6-O21#3	120.04(18)
Cd5-O11	2.186(4)	O1-Cd5-O10	79.98(14)
Cd5-O8	2.331(4)	O1-Cd5-O8	79.81(16)
Cd5-O22#4	2.255(4)	O1-Cd5-O22#4	91.79(17)
Cd5-O9	2.265(5)	O1-Cd5-O9	97.3(2)
Cd1-O17	2.344(4)	O11-Cd5-O10	86.33(16)
Cd1-O29#2	2.329(4)	O11-Cd5-O1	166.28(16)
Cd1-O16	2.200(4)	O11-Cd5-O8	99.02(16)
Cd1-O19	2.240(4)	O11-Cd5-O22#4	91.63(18)
Cd1-O28#2	2.407(4)	O11-Cd5-O9	96.3(2)
Cd1-N4#5	2.334(6)	O8-Cd5-O10	88.94(14)
Cd4-O10	2.357(4)	O22#4-Cd5-O10	100.07(16)

Cd4-O23#4	2.245(4)	O22#4-Cd5-O8	166.50(18)
Cd4-O5#6	2.306(4)	O22#4-Cd5-O9	84.9(2)
Cd4-O12	2.187(4)	O9-Cd5-O10	174.3(3)
Cd4-O6#6	2.393(5)	O9-Cd5-O8	85.7(2)
Cd4-N9#5	2.339(6)	O17-Cd1-O28#2	91.62(16)
O3#1-Cd3-O4#1	54.56(13)	O29#2-Cd1-O17	96.17(15)
O3#1-Cd3-O13#2	104.97(15)	O29#2-Cd1-O28#2	55.11(15)
O3#1-Cd3-O14#2	132.24(16)	O29#2-Cd1-N4#5	82.15(19)
O27-Cd3-O3#1	94.52(14)	O16-Cd1-O17	94.83(16)
O27-Cd3-O4#1	139.81(14)	O16-Cd1-O29#2	143.41(16)
O27-Cd3-O13#2	98.10(15)	O16-Cd1-O19	119.63(17)
O27-Cd3-O14#2	128.83(17)	O16-Cd1-O28#2	89.84(16)
O27-Cd3-N2	86.29(17)	O16-Cd1-N4#5	87.22(18)
O13#2-Cd3-O4#1	113.30(16)	O19-Cd1-O17	85.01(16)
O14#2-Cd3-O4#1	90.44(16)	O19-Cd1-O29#2	96.05(16)
O14#2-Cd3-O13#2	56.10(15)	O19-Cd1-O28#2	150.50(16)
N2-Cd3-O3#1	115.27(16)	O19-Cd1-N4#5	94.0(2)
N2-Cd3-O4#1	85.76(16)	N4#5-Cd1-O17	177.95(16)
N2-Cd3-O13#2	139.08(16)	N4#5-Cd1-O28#2	88.4(2)
N2-Cd3-O14#2	89.67(17)	O10-Cd4-O6#6	102.35(17)
O3#1-Cd2-O17	87.41(14)	O23#4-Cd4-O10	97.70(16)
O18-Cd2-O3#1	100.15(15)	O23#4-Cd4-O5#6	88.45(17)
O18-Cd2-O17	85.40(16)	O23#4-Cd4-O6#6	139.34(17)
O18-Cd2-O26	168.83(16)	O23#4-Cd4-N9#5	85.04(19)
O18-Cd2-O24	96.18(18)	O5#6-Cd4-O10	94.54(16)
O18-Cd2-O15	91.76(18)	O5#6-Cd4-O6#6	55.22(15)
O26-Cd2-O3#1	80.45(14)	O5#6-Cd4-N9#5	97.52(19)
O26-Cd2-O17	83.48(14)	O12-Cd4-O10	82.35(16)
O24-Cd2-O3#1	87.02(17)	O12-Cd4-O23#4	118.56(18)
O24-Cd2-O17	174.40(17)	O12-Cd4-O5#6	152.99(17)
O12-Cd4-N9#5	85.81(18)	O12-Cd4-O6#6	99.05(16)
N9#5-Cd4-O10	167.70(17)	N9#5-Cd4-O6#6	82.8(2)

Symmetry transformations used to generate equivalent atoms: #1 -1+X, +Y,

+Z; #2 +X,1/2-Y,-1/2+Z; #3 1+X,1/2-Y,1/2+Z; #4 1+X,+Y, +Z; #5 +X,-1+Y,+Z; #6 +X,1/2-Y,1/2+Z; #7 -1+X,1/2-Y,-1/2+Z; #8 +X,1+Y,+Z.

**2**			
Cu(2)-Cu(1)	2.8159(13)	Cu(2)-O(7)	2.068(6)
Cu(1)-O(6)	1.994(6)	Cu(2)-O(9)#1	2.026(6)
Cu(1)-O(10)#1	2.042(6)	Cu(2)-O(5)#2	2.068(6)
Cu(1)-O(4)#2	2.038(6)	Cu(2)-O(2)	2.042(7)
Cu(1)-O(1)	2.004(6)	Cu(2)-N (3)	2.166(8)
O(6)-Cu(1)-Cu(2)	84.22(16)	O(1)-Cu(1)-N(1)	95.6(3)
O(6)-Cu(1)-O(10)#2	89.5(3)	N(1)-Cu(1)-Cu(2)	175.1(2)
O(6)-Cu(1)-O(4)#1	88.1(3)	O(7)-Cu(2)-Cu(1)	78.87(17)
O(6)-Cu(1)-O(1)	164.6(2)	O(7)-Cu(2)-O(5)#1	87.6(3)
O(6)-Cu(1)-N(1)	99.8(3)	O(7)-Cu(2)-N(3)	96.0(3)
O(10)#2-Cu(1)-Cu(2)	83.37(16)	O(9)#2-Cu(2)-Cu(1)	80.65(15)
O(10)#2-Cu(1)-N(1)	93.7(3)	O(9)#2-Cu(2)-O(7)	89.5(3)
O(4)#1-Cu(1)-Cu(2)	80.11(17)	O(9)#2-Cu(2)-O(5)#1	164.4(2)
O(4)#1-Cu(1)-O(10)#2	163.5(2)	O(9)#2-Cu(2)-O(2)	88.0(3)
O(4)#1-Cu(1)-N (1)	102.8(3)	O(9)#2-Cu(2)-N(3)	100.5(3)
O(1)-Cu(1)-Cu(2)	80.39(16)	O(5)#1-Cu(2)-Cu(1)	83.72(17)
O(1)-Cu(1)-O(10)#2	88.5(3)	O(5)#1-Cu(2)-N(3)	95.0(3)
O(1)-Cu(1)-O(4)#1	89.5(3)	N(3)-Cu(2)-Cu(1)	174.7(2)

Symmetry transformations used to generate equivalent atoms: #1 3/2-X,1/2-Y, -1/2+Z; #2 3/2-X,3/2-Y,1/2+Z

Table S2. Standard deviation and detection limit calculation of **1** for Glu and Asp in aqueous suspension.

	Glu	Asp
1	788.5851	839.4675
2	788.4088	839.6109
3	788.7129	838.8484
Standard deviation (σ)	0.1247	0.3309
K_{sv}	8.43×10^3 M^{-1}	9.74×10^3 M^{-1}
Detection limit (3σ/ K_{sv})	4.44×10^{-5} M	1.02×10^{-4} M

Table S3. Fluorescence lifetime of MOF **1** before and after addition of Glu and Asp.

AAs	MOF **1**		MOF **1** + AA	
	Lifetime (ns)	R^2	Lifetime (ns)	R^2
Glu	20.4	0.99911	26.7	0.99911
Asp	19.6	0.99943	27.2	0.99925

Table S4. H_2O_2-assisted photocatalytic degradation of aqueous RhB by MOF **2** and reported MOFs under visible light.

MOFs	Efficiency (%)	T (min)	k (min^{-1})	Ref.
$[Cu_2(DPA)(OBA)_2]$ (**2**)	99	120	0.0375	This work
$Co_3(BPT)_2(bpp)$	90	120	0.0192	[3]
CoFe-MOF	97	100	0.02257	[4]
$Zn_3(BTC)_2(H_2O)_3$	85	85	0.0095	[5]
NNU-36	96	70	0.0468	[6]

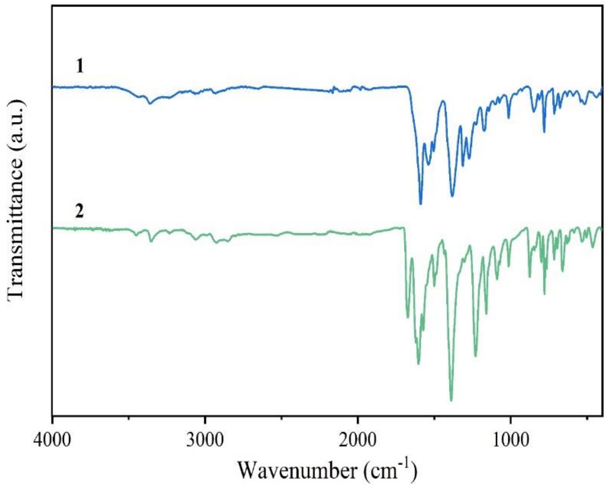

Figure S1. FTIR-ATR spectra of **1** and **2**.

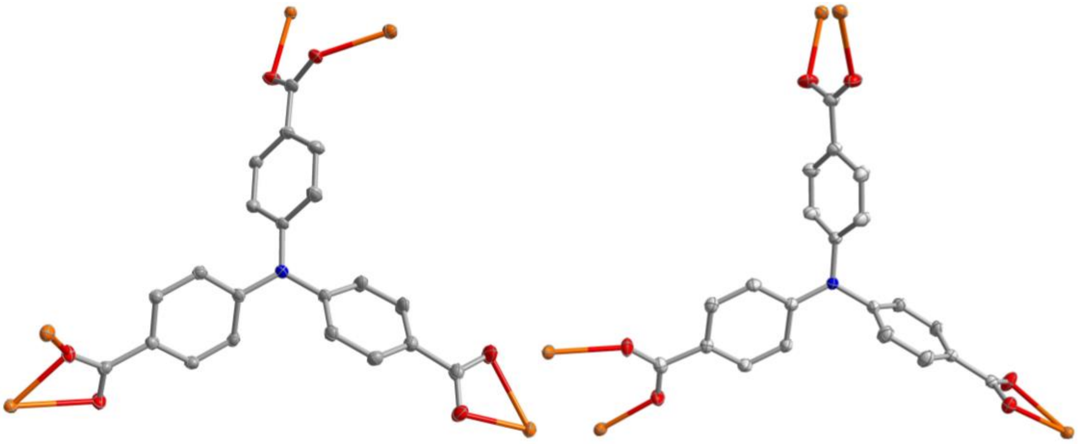

(a) (b)

Figure S2. Coordination modes of NTB^{3-} in **1**.

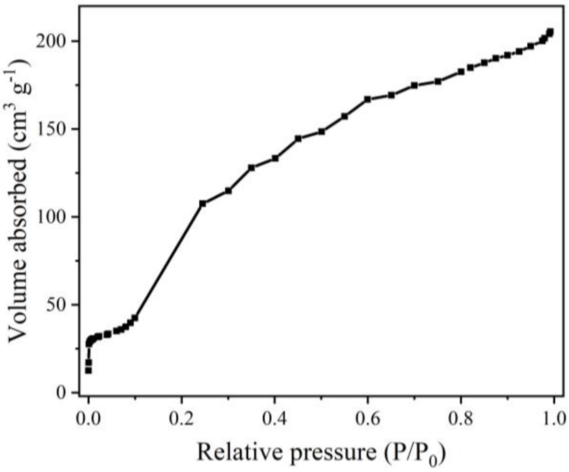

Figure S3. The N_2 adsorption isotherm of MOF **2** at 77 K.

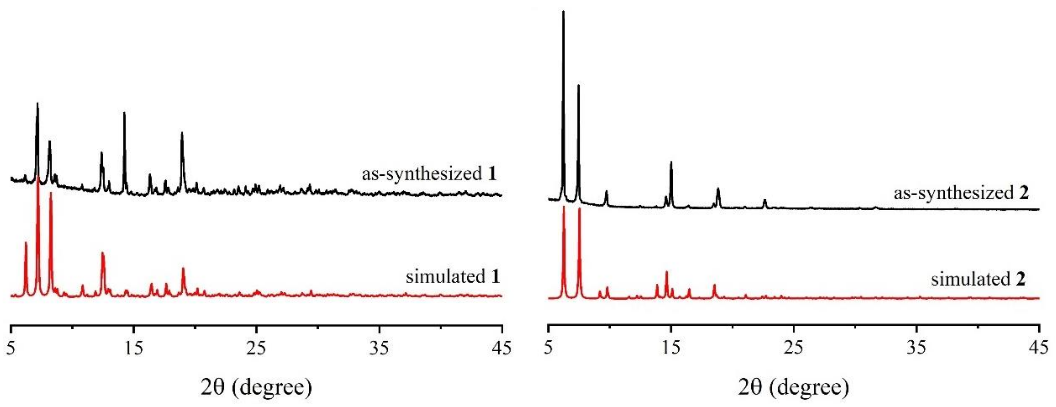

(a) (b)

Figure S4. PXRD patterns of **1** and **2**.

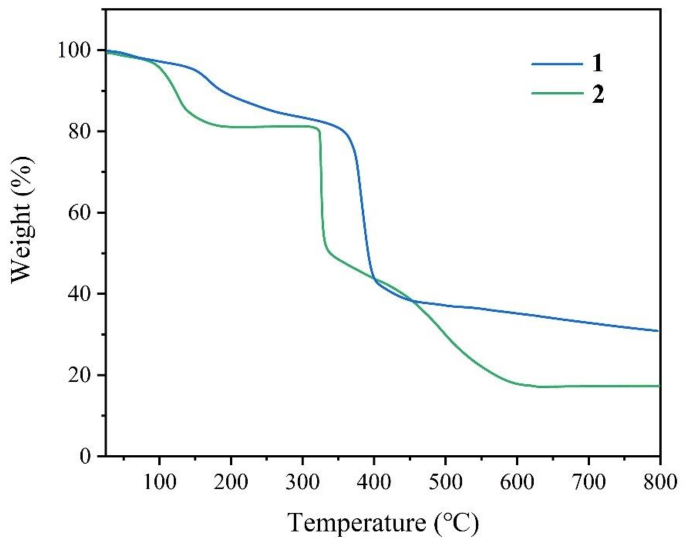

Figure S5. TG curves of **1** and **2**.

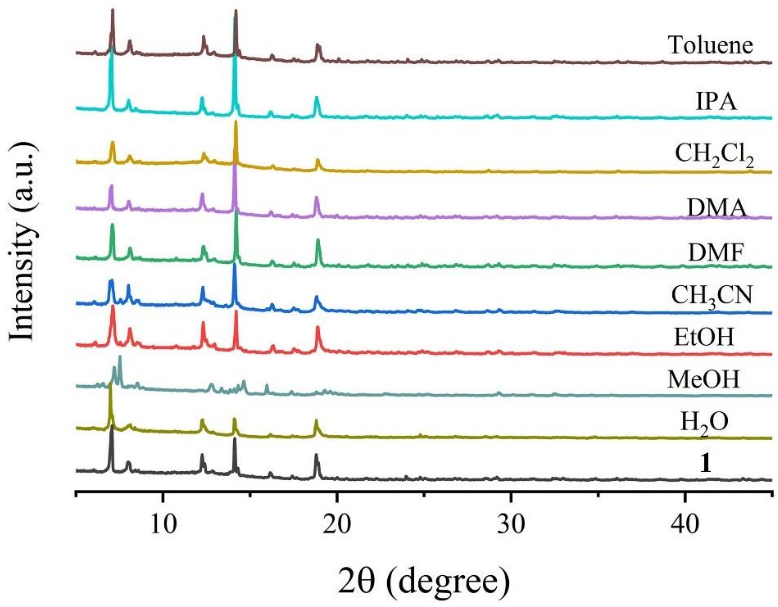

Figure S6. PXRD of **1** after immerging in different solvent.

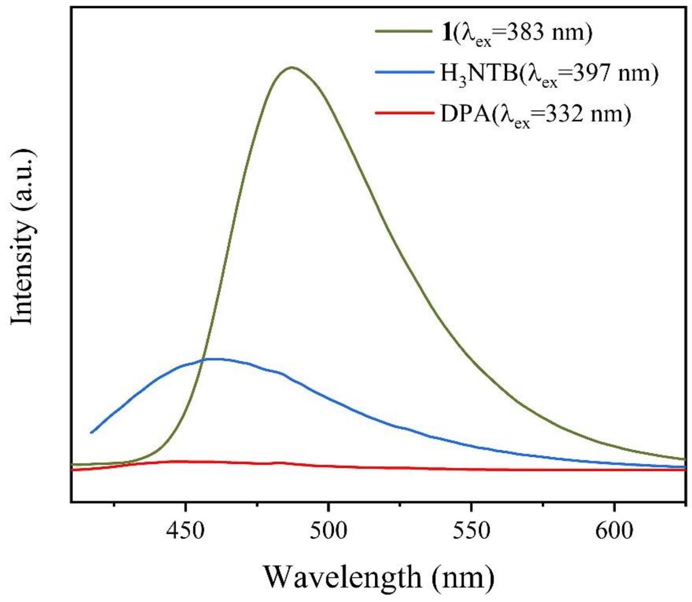

Figure S7. Fluorescence spectra of MOF **1** and its ligands H_3NTB and DPA in the solid state.

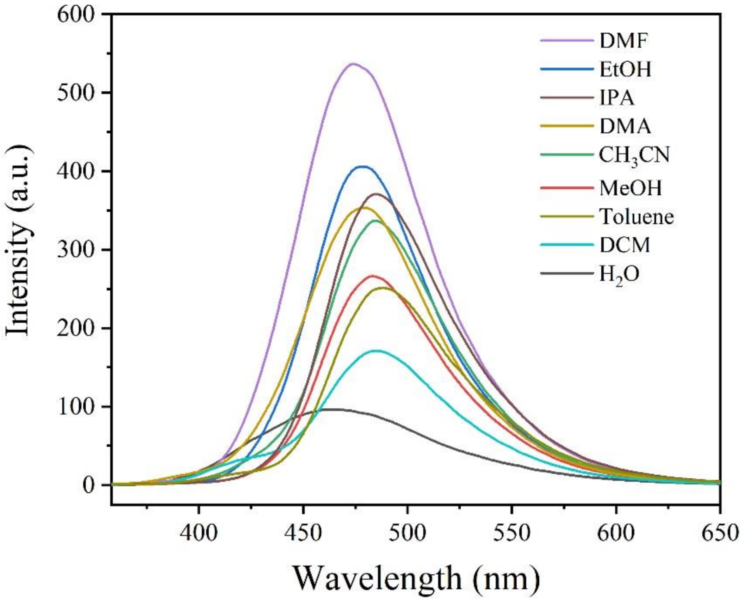

Figure S8. PL spectra of **1** after immerging in varied solvent.

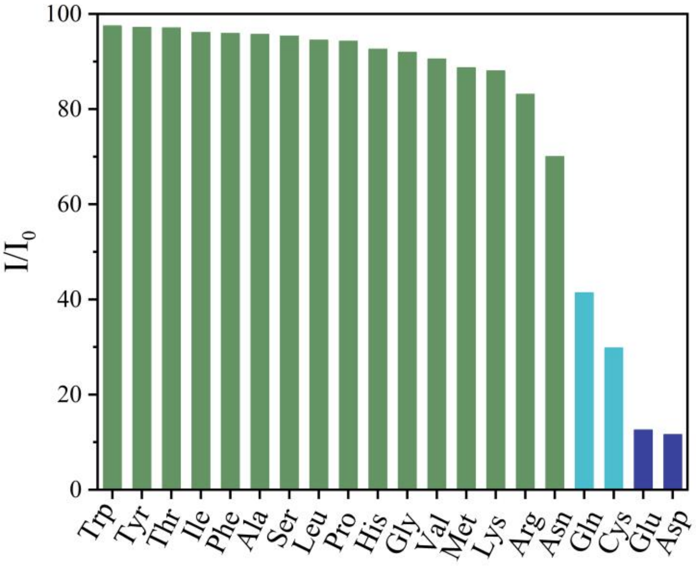

Figure S9. Fluorescence quenching effect of **1** by adding amino acid aqueous solution (300 μL, 2.5 mM, λ_{ex} = 338 nm).

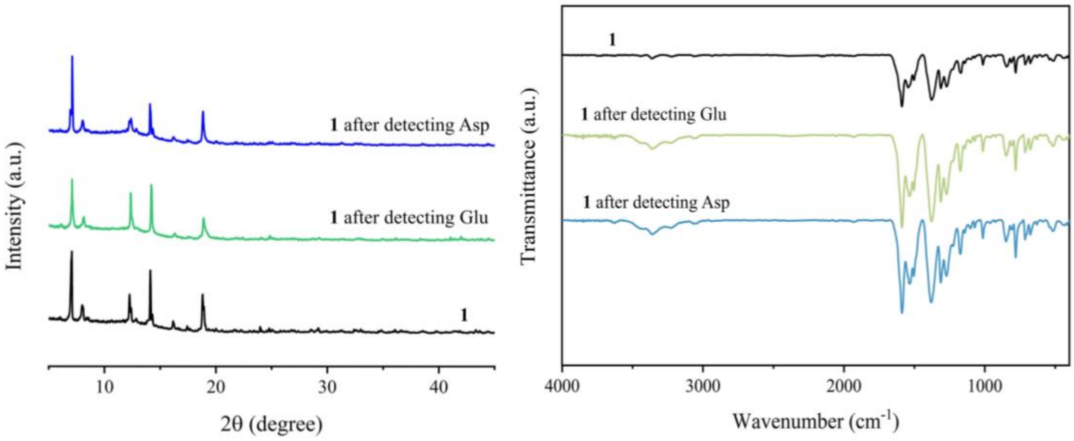

(a) (b)

Figure S10. PXRD (a) and FTIR-ATR (b) spectra of MOF **1** before and after detecting Glu and Asp.

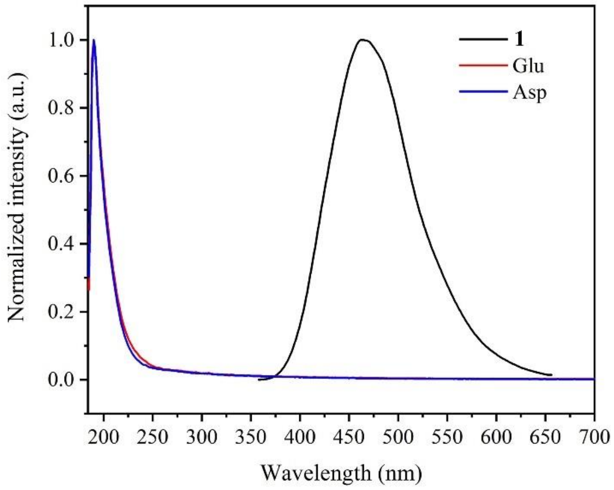

Figure S11. Fluorescence emission spectra of **1** and UV absorption spectra of Glu and Asp in water.

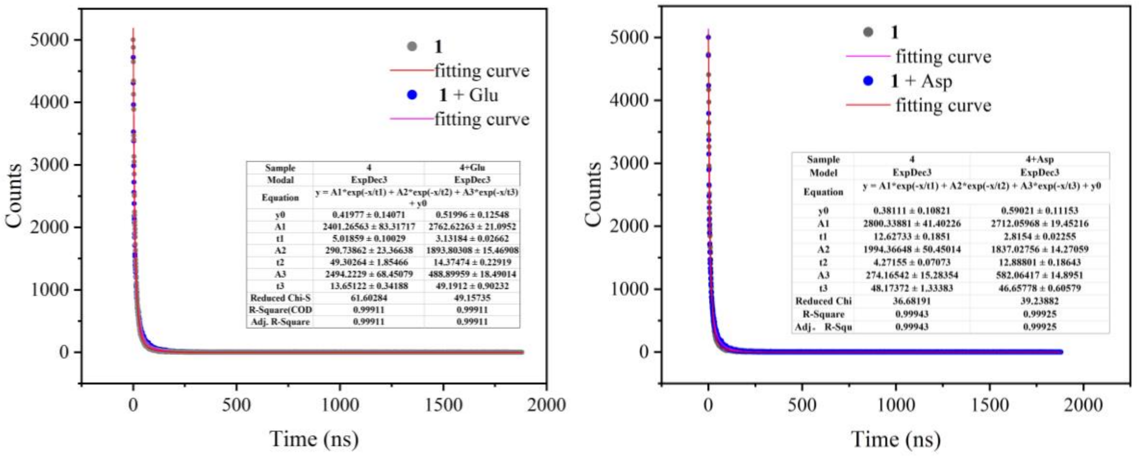

(a) (b)

Figure S12. Fluorescence lifetime of **1** before and after detecting Glu (a) and Asp (b) in water.

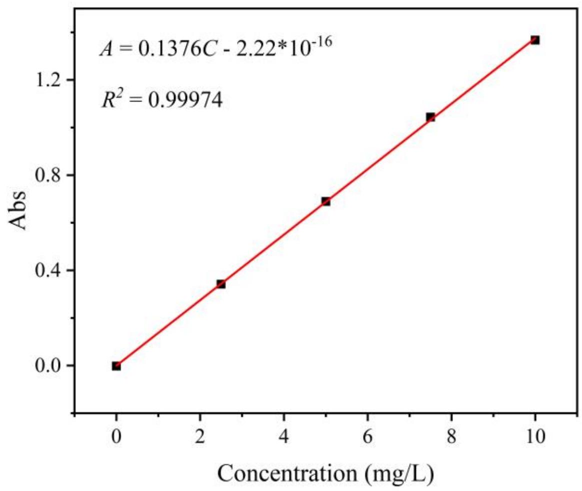

Figure S13. The standard curve of MOF **2** for the UV absorption versus concentration of RhB.

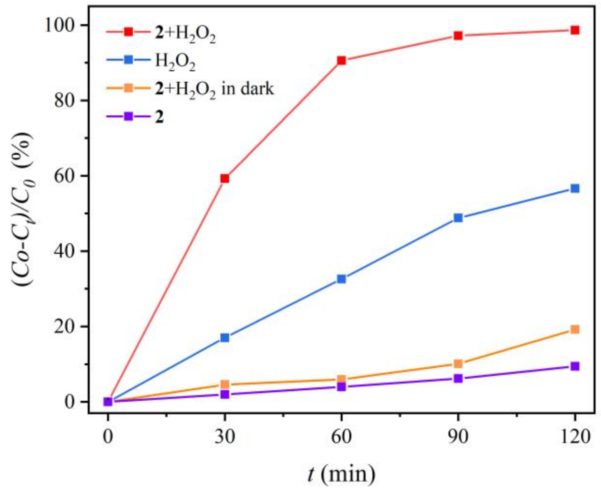

Figure S14. The degradation efficiency of RhB by **2** at varied reaction time.

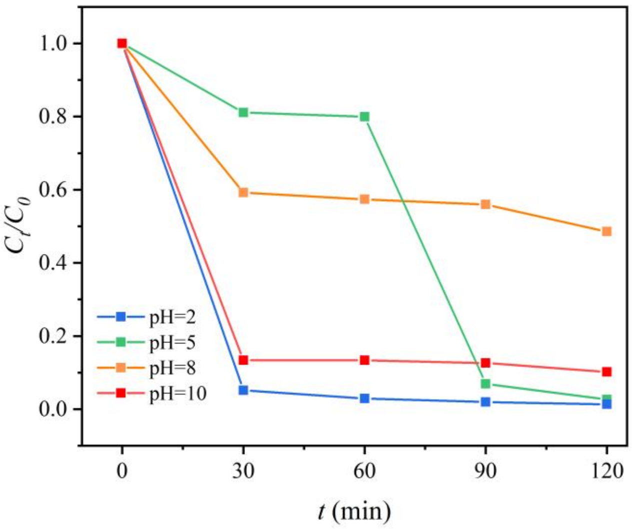

Figure S15. The concentration of RhB in the degradation process at acidic (pH = 2 and 5) and basic (pH = 8 and 10) conditions.

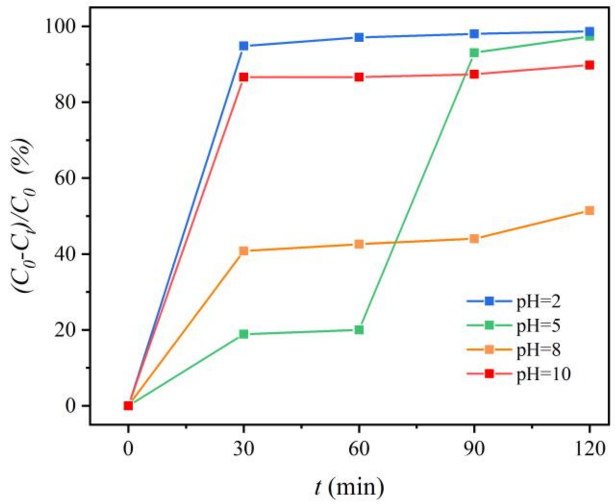

Figure S16. The degradation efficiency of RhB by **2** at acidic (pH = 2 and 5) and basic (pH = 8 and 10) conditions.

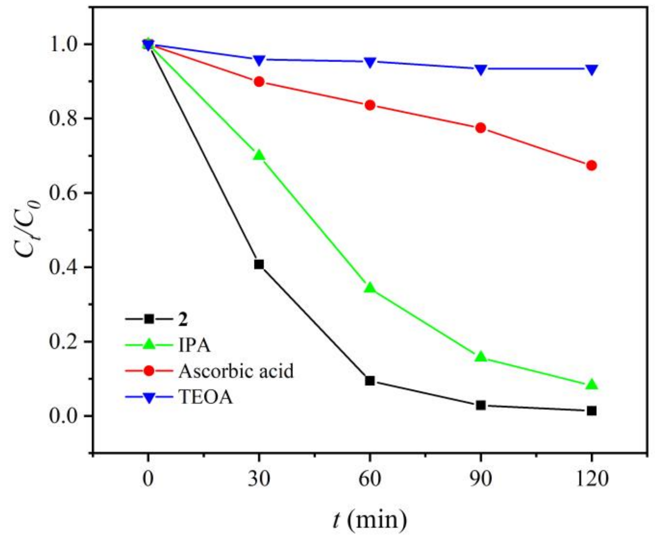

Figure S17. The concentration of RhB at varied photodegradation time with different radical trapping agents.

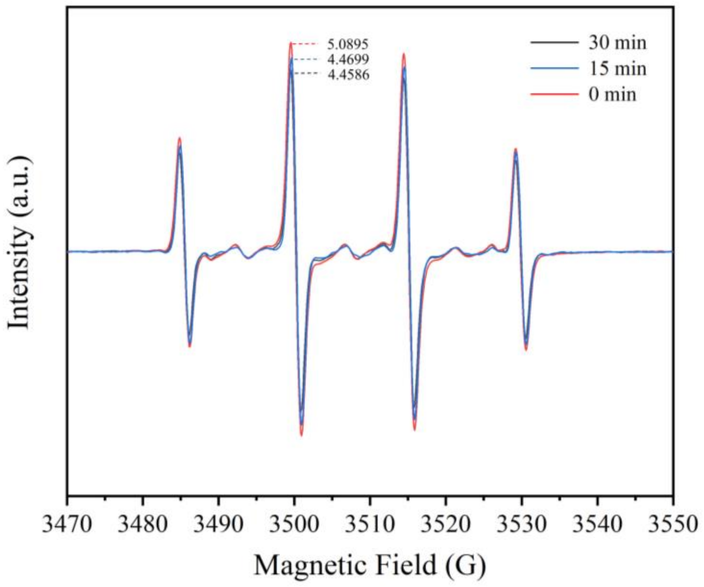

Figure S18. The EPR spectra of photodegradation of RhB by MOF **2** at different reaction time using DMPO as spin-trapping agent.

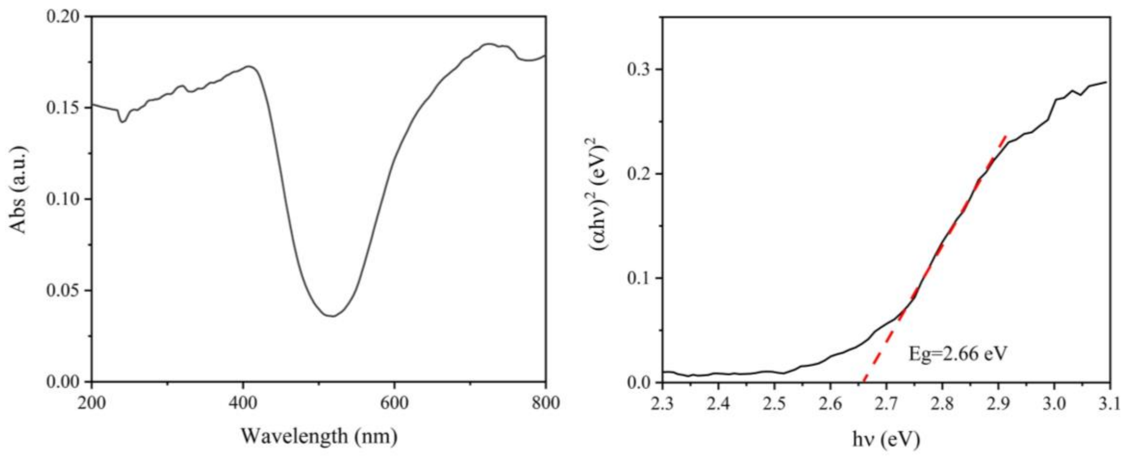

(a) (b)

Figure S19. UV-vis diffuse reflectance spectrum (a) and Tauc plot (b) of MOF **2**.

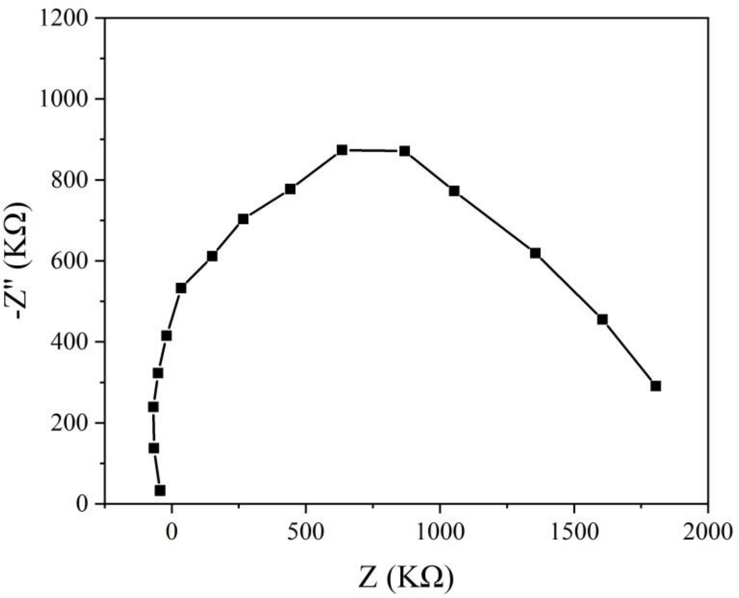

Figure S20. EIS Nyquist plot of MOF **2**.

References

1. Guo, X.G.; Zhang, Z.Y.; Qiu, S.; Su, X.; Wang, Y.B.; Sun, X.Q. Versatile Tailoring of NH_2-Containing Metal–Organic Frameworks with Paddle-Wheel Units. *Chem. Eur. J.* **2017**, *23*, 17727–17733.
2. Yang W., Wang J., Wang H., Bao Z., Zhao J. C.-G. and Chen B., Highly Interpenetrated Robust Microporous Hydrogen-Bonded Organic Framework for Gas Separation. *Cryst. Growth Des.*, **2017**, *17*, 6132-6137.
3. Xia Q., Yu X., Zhao H., Wang S., Wang H., Guo Z. and Xing H., Syntheses of Novel Lanthanide Metal–Organic Frameworks for Highly Efficient Visible-Light-Driven Dye Degradation. *Cryst. Growth Des.*, **2017**, *17*, 4189-4195.
4. Tran T. K. N., Ho H. L., Nguyen H. V., Tran B. T., Nguyen T. T., Bui P. Q. T. and Bach L. G., Photocatalytic Degradation of Rhodamine B in Aqueous Phase by Bimetallic Metal-Organic Framework M/Fe-MOF (M = Co, Cu, and Mg). *Open Chemistry*, **2022**, *20*, 52-60.
5. Sarkar A., Adhikary A., Mandal A., Chakraborty T. and Das D., Zn-BTC MOF as an Adsorbent for Iodine Uptake and Organic Dye Degradation. *Cryst. Growth Des.*, **2020**, *20*, 7833-7839.
6. Zhao H., Xia Q., Xing H., Chen D. and Wang H., Construction of Pillared-Layer MOF as Efficient Visible-Light Photocatalysts for Aqueous Cr(VI) Reduction and Dye Degradation. *ACS Sustain. Chem. Eng.*, **2017**, *5*, 4449-4456.
